# Discovery of small molecule inhibitors that effectively disrupt IQGAP1-Cdc42 interaction in breast cancer cells

**DOI:** 10.1038/s41598-022-21342-w

**Published:** 2022-10-17

**Authors:** Samar Sayedyahossein, Jessica Smith, Elena Barnaeva, Zhigang Li, Jun Choe, Michael Ronzetti, Christopher Dextras, Xin Hu, Juan Marugan, Noel Southall, Bolormaa Baljinnyam, Louise Thines, Andy D. Tran, Marc Ferrer, David B. Sacks

**Affiliations:** 1 Department of Laboratory Medicine, National Institutes of Health, Bethesda, MD 20892 USA; 2 National Center for Advancing Translational Sciences, National Institutes of Health, Rockville, MD USA; 3 Confocal Microscopy Core Facility, Laboratory of Cancer Biology and Genetics, NCI, National Institutes of Health, Rockville, MD USA; 4 Present Address: Department of Physiology and Pharmacology, University of Western Ontario, London, ON Canada; 5 Present Address: Center for Scientific Review, National Institutes of Health, Bethesda, MD USA

**Keywords:** Biochemistry, Cell biology

## Abstract

The small GTPase Cdc42 is an integral component of the cytoskeleton, and its dysregulation leads to pathophysiological conditions, such as cancer. Binding of Cdc42 to the scaffold protein IQGAP1 stabilizes Cdc42 in its active form. The interaction between Cdc42 and IQGAP1 enhances migration and invasion of cancer cells. Disrupting this association could impair neoplastic progression and metastasis; however, no effective means to achieve this has been described. Here, we screened 78,500 compounds using a homogeneous time resolved fluorescence-based assay to identify small molecules that disrupt the binding of Cdc42 to IQGAP1. From the combined results of the validation assay and counter-screens, we selected 44 potent compounds for cell-based experiments. Immunoprecipitation and cell viability analysis rendered four lead compounds, namely NCGC00131308, NCGC00098561, MLS000332963 and NCGC00138812, three of which inhibited proliferation and migration of breast carcinoma cells. Microscale thermophoresis revealed that two compounds bind directly to Cdc42. One compound reduced the amount of active Cdc42 in cells and effectively impaired filopodia formation. Docking analysis provided plausible models of the compounds binding to the hydrophobic pocket adjacent to the GTP binding site of Cdc42. In conclusion, we identified small molecules that inhibit binding between Cdc42 and IQGAP1, which could potentially yield chemotherapeutic agents.

## Introduction

The 189-kDa scaffold protein, IQGAP1, contains several protein-interaction domains, namely a calponin homology domain (CHD), a polyproline-binding (WW) domain, four calmodulin-binding IQ motifs (IQ), and a Ras-GAP-related domain (GRD)^1,2^. The multiple domains of IQGAP1 enable it to interact with a wide variety of signaling and structural proteins, such as actin, calmodulin, members of the Rho GTPase family (e.g. Rac1 and Cdc42), β-catenin, components of the phosphoinositide 3-kinase (PI3K) /AKT pathway, and adenomatous polyposis coli^2,3^. Through its plethora of binding partners, IQGAP1 regulates multiple fundamental cellular processes, including cytoskeletal organization, cell–cell adhesion, cell migration, transcription, and signal transduction^2,4^.

The contribution of IQGAP1-mediated signaling to different stages of cancer progression is an emerging field^2,5^. IQGAP1 is overexpressed in numerous human cancer cell lines and tissues^5^. We previously documented that overexpression of IQGAP1 in human breast epithelial cells enhances tumor proliferation, invasion, and angiogenesis^6^. IQGAP1 is an oncogene that is not required for cell homeostasis^7^, making it an attractive molecule for the development of targeted therapies. Published data imply that the interaction of IQGAP1 with selected binding partners can be specifically targeted to attenuate neoplastic processes. For example, treatment of mice with cell-permeable peptides that disrupt IQGAP1-extracellular signal regulated kinase (ERK)1/2 interactions inhibit Ras driven tumorigenesis^8^.

Cdc42 is among the best characterized IQGAP1 binding partners^2^. Cdc42 is a Rho GTPase that is important for neoplastic transformation of cells by Ras and other oncoproteins^9^. Rho GTPases oscillate between a GTP-bound active form and a GDP-bound inactive state^10^. Guanine nucleotide exchange factors (GEFs) promote the exchange of GDP for GTP, whereas GTPase activating proteins (GAPs) enhance the intrinsic GTPase activity of the small G proteins, promoting hydrolysis of the bound GTP. Importantly, IQGAP1 does not have GAP activity^11^. Instead, when IQGAP1 binds to the GTP-bound (active) form of Cdc42, it inhibits Cdc42 intrinsic GTPase activity, keeping Cdc42 active^12,13^. A constitutively active Cdc42 mutant construct, termed Cdc42-Q61L, binds IQGAP1with high affinity^14^. In contrast, when amino acid residue N17 in Cdc42 is mutated, the construct fails to bind to many Cdc42 binding partners, including IQGAP1^14^.

Cdc42 is not mutated in cancers; instead, dysregulation of Cdc42 functions contribute to carcinogenesis^9^. Increased levels of active Cdc42 directly impact actin cytoskeletal dynamics and filopodia formation^15,16^, which are crucial for cell migration, adhesion and responses to external stimuli^17^. Importantly, overexpression of IQGAP1 increases the pool of active Cdc42 in the cells, while knockdown of endogenous IQGAP1 decreases the amount of active Cdc42^13^. Moreover, Cdc42 is an important component of IQGAP1-mediated cell proliferation, tumorigenesis, and invasion^6^. Therefore, blocking the formation of IQGAP1/Cdc42 complexes using small molecule inhibitors could reduce the amount of active Cdc42 in malignant cells, thus reducing tumorigenesis.

In this study, we used a high throughput screening (HTS) approach to identify small molecules that interfere with IQGAP1/Cdc42 interactions. We screened approximately 78,500 small molecules using a homogeneous time resolved fluorescence (HTRF) assay and identified four compounds that interfere with IQGAP1/Cdc42 interactions and can potentially be used in targeted therapies.

## Results

### Development of the HTRF assay

**Figure 1:**
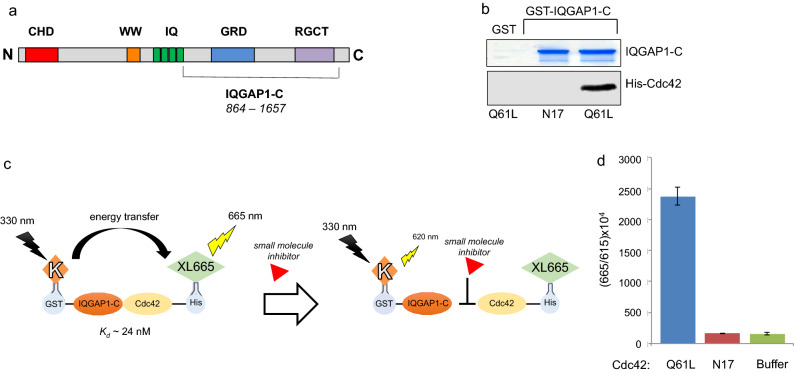
Detection of IQGAP1/Cdc42 complex formation. (**a**) Schematic representation of IQGAP1 protein. The C-terminal half of IQGAP1 (amino acids 864–1657) was used in the HTRF assay. (**b**) GST pull-down assay. GST-IQGAP1-C was incubated with His-tagged Cdc42-N17 or Cdc42-Q61L for 2 h at 4 °C. GST alone was incubated with Cdc42-Q61L as the negative control. Complexes were isolated using glutathione-Sepharose and resolved by SDS-PAGE. The gel was cut at 70 kDa. The upper part of the gel was stained with Coomassie blue. The lower portion of the gel was transferred to PVDF membrane and probed with anti-Cdc42 antibodies. (**c**) Scheme of the HTRF assay in 384–well format showing fluorescent labeled GST-IQGAP1-C (donor Europium cryptate, K) and His-Cdc42-Q61L (acceptor XL665). (**d**) Time resolved fluorescence was detected at 615 and 665 nm on the Biotek Synergy using recombinant proteins. The Y-axis represents the ratio of acceptor to donor emission wavelength multiplied by 10,000. The means and standard errors of two replicates are shown. The green bar (Buffer) indicates samples without His-Cdc42.

In order to identify compounds that disrupt the interaction between IQGAP1 and Cdc42, we developed a HTRF-based assay and adapted it to a 1536-well plate format to enable HTS. We used the glutathione S-transferase (GST)-tagged C-terminal half (amino acids 864–1657) of IQGAP1 (Fig. 1a) and hexa-histidine (His)-tagged Cdc42-Q61L, because of their high binding affinity.

We previously reported that constitutively active Cdc42-Q61L binds to this fragment of IQGAP1 with an equilibrium dissociation constant (K_d_) of 24 ± 4 nM measured by scintillation proximity assay^14^. In agreement with our previous observations^14^, analysis by GST pulldown and Western blotting revealed that Cdc42-Q61L binds to the C-terminal region of IQGAP1 (Fig. 1b). In contrast, dominant negative Cdc42-N17 has no detectable binding to IQGAP1 (Fig. 1b). In the HTRF assay, the binding of Cdc42 to IQGAP1 was detected using anti-6xHis-XL665 and anti-GST-cryptate antibodies. When Cdc42-Q61L is bound to IQGAP1-C, the corresponding antibodies come into close proximity, initiating energy transfer between the long-life fluorescent Europium^3+^ cryptate donor and the XL665 acceptor (Fig. 1c). The energy is emitted as a detectable fluorescent signal at 665 nm, which is proportional to the level of interaction. When a molecule interrupts the binding, the proximity of antibodies will not be adequate to produce energy transfer, and the signal will be reduced or absent, resulting in a diminished fluorescent signal (Fig. 1c).

We initially evaluated the HTRF assay in 384-well-plate format and used Cdc42-N17 as the negative control. A robust fluorescence signal was detected when IQGAP1-C was incubated with Cdc42-Q61L (Fig. 1d). By contrast, when IQGAP1-C was incubated with Cdc42-N17, the fluorescence was minimal, essentially the same as that observed in the absence of Cdc42-Q61L (IQGAP1 only samples). These data validate that we can detect a specific interaction between Cdc42 and IQGAP1 in a multiwell plate format, enabling us to use this assay to screen for small molecule inhibitors by HTRF.

The assay was miniaturized and optimized to a 1536-well-plate format (see Methods for details). Then, the Library of Pharmacologically Active Compounds (LOPAC®^1280^, Sigma Aldrich) collection was screened in dose response to evaluate the robustness of the assay in a screening format and in the presence of compounds. The compounds were tested at seven concentrations. The final concentrations of the compounds in the assay ranged from 38 µM to 3.6 nM. HTRF signals in the presence of DMSO and 1.5 nM IQGAP1 protein alone were collected for each plate and set as IC_0_ and IC_100_, respectively. The median assay parameters were 7.1-fold signal-to-background ratio (S/B) and 0.87 Z’-factor, indicating a robust assay performance amenable to HTS. We conducted a DMSO tolerability assay using 4% DMSO to assess the potential effect of DMSO on the HTRF signal. The data confirmed that DMSO alone does not alter the HTRF signal (See Supplementary Fig. S1 online).

### High throughput screening and counter-screens

**Figure 2:**
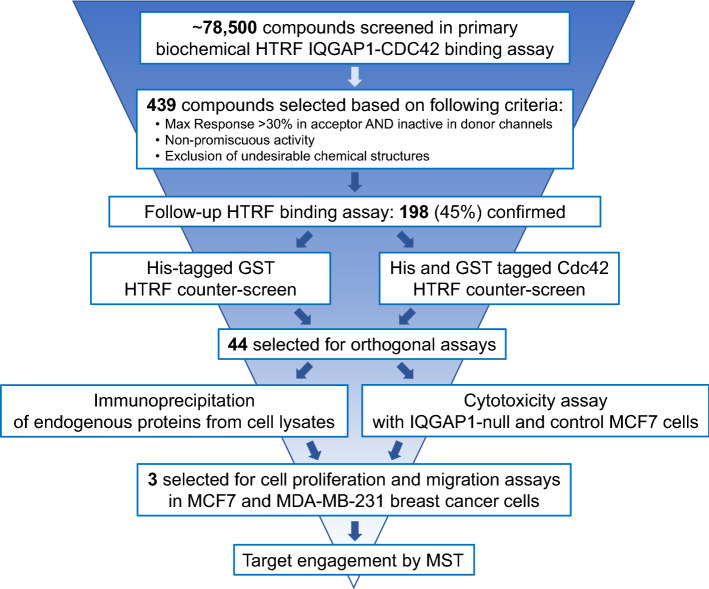
Compound identification and validation flow chart. Depicted is the funnel of the assays, filtering, and analyses performed for identification of the most prominent chemotypes of clusters and singletons selected for further studies.

We screened approximately 78,500 small molecules using the primary HTRF assay (Fig. 2). Each compound was tested at four doses, with concentrations ranging from 38 µM to 305 nM. Primary hits were selected based on the following criteria: (1) maximum response > 30% of the signal on the scale between vehicle control DMSO (IC_0_) and GST-IQGAP1-C only as maximal potential inhibition (IC_100_), (2) inactivity in the cryptate donor channel (i.e., no fluorescence) and (3) lack of promiscuous and/or undesirable chemical structure (dopamine-like, quinolone-like, thiourea, etc.). Based on these criteria, 439 compounds were selected for further analysis (Fig. 2).

The compounds were re-tested in the same primary HTRF IQGAP1-Cdc42 binding assay at seven doses, in the concentration range of 38 μM to 50 nM. One hundred and ninety-eight (~ 45%) of the 439 primary hits were confirmed in the validation screening.

Conceivably, some of the compounds identified in the HTRF assay could be artifacts, i.e., compounds that interfere with the HTRF assay technology or antibody-tag binding. To eliminate these false positives, we tested the compounds in two different HTRF counter-screens. The counter-screens were similar to the primary HTRF assay, but instead of the GST-IQGAP1 and His-Cdc42 proteins, a hexa-histidine tagged GST (His-GST) or a double-tagged Cdc42-Q61L containing GST at the N-terminus and hexa-histidine at the C-terminus (GST-Cdc42-Q61L-His) were used. Ninety-nine compounds were identified as false positives based on these counter-screens. Interestingly, some of the compounds did not show any activity in the His-GST counter-screen but were active in the counter-screen with the double-tagged Cdc42-Q61L.

### Evaluation of the hit compounds in orthogonal assays

Based on the validation and counter screening data, we selected 44 compounds using compound dose response curve algorithms developed at NCATS^18^ for orthogonal assays in cell lysates and breast cancer cell lines (Fig. 2). These were the most potent compounds (EC_50_ < 15 µM) representing the largest structural clusters.

**Figure 3:**
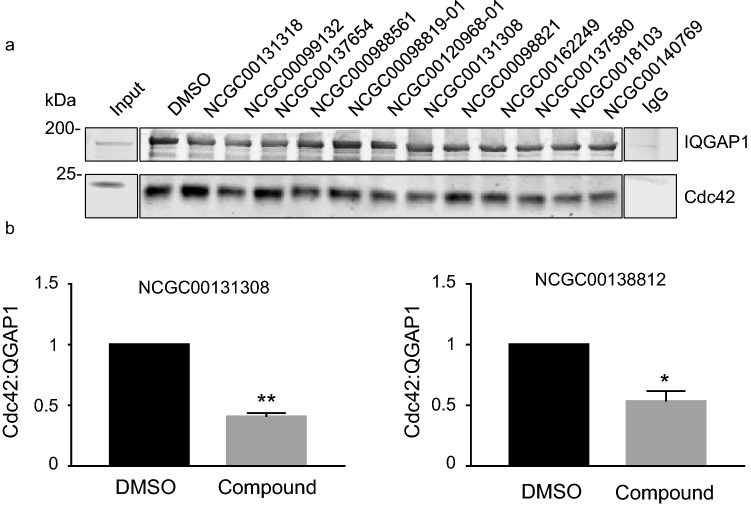
Compounds that impair the IQGAP1/Cdc42 interaction in cell lysates. (**a**) HEK293 cell lysates were prepared as described in the Methods section. The selected compounds (50uM) or DMSO (vehicle) were added to equal aliquots of protein lysates and endogenous IQGAP1 was immunoprecipitated with polyclonal anti-IQGAP1 antibodies. Anti-rabbit IgG was used as the negative control (last lane). Immune complexes were analyzed by SDS-PAGE and transferred to PVDF membrane. Western blots were probed with anti-IQGAP1 and anti-Cdc42 antibodies. 1% of the protein lysates was loaded directly onto the gel (Input). The data are representative of at least three independent experiments. Vertical lines indicate where irrelevant lanes were removed from the blots and added sections from a separate blot. Full-length blots are presented in Supplementary Fig. 5. (**b**) The IQGAP1 and Cdc42 bands were quantified with Image Studio 2.0 (LI-COR Biosciences) and the amount of Cdc42 was corrected for IQGAP1 in the same sample. Data from 16 µM NCGC00131308 to 0.2 µM NCGC00138812 are shown. The data are expressed as means ± SEM (error bars) with DMSO set to 1 (n = 3). Statistical analysis was conducted using paired *Student’s t-test,* * *p* < 0.05 and ** *p* < 0.01 compared to control.

Immunoprecipitation was used to examine the ability of the compounds to interrupt the interaction between native and full-length Cdc42 and IQGAP1 proteins. Each compound was added to lysates from HEK293 cells at a final concentration of 50 µM and endogenous IQGAP1 was immunoprecipitated. DMSO alone was used as the negative control (100% binding). Samples were analyzed by Western blotting and blots were probed for IQGAP1 and Cdc42. The amount of endogenous Cdc42 that co-immunoprecipitated with IQGAP1 was quantified and normalized to the amount of IQGAP1 immunoprecipitated from the same sample. A representative blot with 12 of the 44 evaluated compounds is shown in Fig. 3a.

No Cdc42 co-precipitated with rabbit IgG, documenting the specificity of the immunoprecipitation. The compounds were tested in at least three independent experiments and the most promising compounds were re-tested at different concentrations (0.2, 0.6, 1, 5, 16 or 50 µM). Four of the 44 compounds, namely NCGC00131308, NCGC00098561, MLS000332963 and NCGC00138812, reduced the binding substantially (Table 1). NCGC00131308 and NCGC00138812 significantly reduced the interaction between endogenous IQGAP1 and Cdc42 by a mean of 60% and 47%, respectively (Fig. 3b).

**Table 1:** Structures and in vitro physicochemical properties of four selected compounds, NCGC00131308, NCGC00138812, MLS000332963 and NCGC0009856, with notable activity in immunoprecipitation assay.

ID	Structure	T_1/2_ (min)^a^	PAMPA (1e-6 cm/s)^b^	Solubility (µg/mL)^c^
NCGC00131308		2.7	77	37.15
NCGC00138812		7.12	177	< 1
MLS000332963		11.47	675	< 1
NCGC00098561		NA	NA	NA

^a^T_1/2_: metabolic half-life measured in rat liver microsome lysates reported in minutes (minimum detectable half-life of 1 min).

^b^Parallel artificial membrane permeation assay (PAMPA) is reported as a metric of the passive permeability of the compounds.

^c^Solubility–pION µSOL assay for kinetic aqueous solubility determination, pH 7.4.

**Figure 4:**
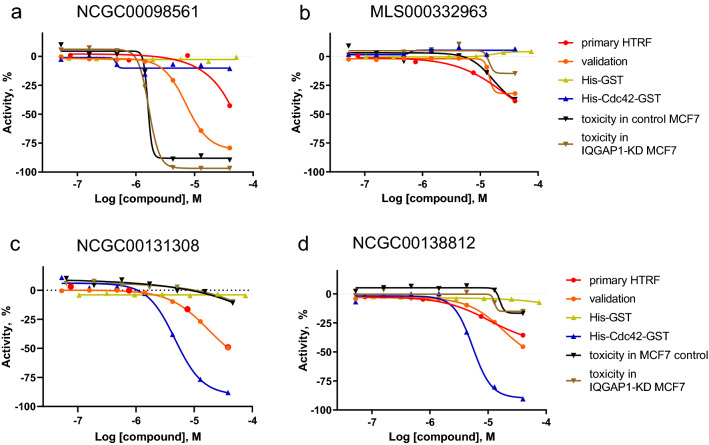
Dose responses of four selected compounds in HTRF, counter-screen and cytotoxicity assays. (**a**) NCGC00098561, (**b**) MLS000332963, (**c**) NCGC00131308 and (**d**) NCGC00138812. Primary HTRF—primary HTRF high-throughput screening; validation—confirmatory HTRF assay; His-GST—counter-screen with His-GST; His-Cdc42-GST—counter-screen with dual-tagged Cdc42; toxicity in control MCF7—cytotoxicity assay in control MCF7 cells; toxicity in IQGAP1-KD MCF7—cytotoxicity assay in IQGAP1-KD MCF7 cells.

In addition, the selected 44 compounds were assessed for their effects on cell viability. Analysis was performed in both MCF7 breast carcinoma cells with stable knockdown of IQGAP1 by siRNA (termed MCF7-siIQ8) and control MCF7 cells (see Methods for details), to decipher whether the cytotoxicity could be caused by the disruption of the target protein–protein-interaction. Out of the four compounds which were active in immunoprecipitation assay, only NCGC00098561 showed a cytotoxicity with an EC_50_ of 2.24 µM and an efficacy of about 100% in both cell lines, suggesting that the toxicity was independent of IQGAP1 (Fig. 4a).

MLS000332963 showed a moderate cytotoxicity (EC50 = 19.95 µM, efficacy of 47%) in control MCF7 cells only (Fig. 4b). The remaining two compounds did not show any noticeable effect on cell viability.

We selected compounds NCGC00131308, NCGC00138812 and MLS000332963 for additional cell-based experiments due to their activities in the primary, counter-screening and orthogonal assays (Fig. 4b–d, Table 1). Interestingly, while all three compounds were inactive in the His-GST counter-screen, NCGC00131308 and NCGC00138812 were active in the double-tagged Cdc42 counter-screen with EC_50_ values of 4.47 and 6.31 µM, respectively. Although NCGC00098561 showed good activity in disrupting IQGAP1 and Cdc42 interaction in the biochemical HTRF assay (validation assay: EC_50_ = 7.94 µM, efficacy 84%) and was not active in either of the counter-screens (Fig. 4a), it was removed from further cell-based experiments because of its cytotoxicity.

### Effects of the lead molecules on cell proliferation and migration

**Figure 5:**
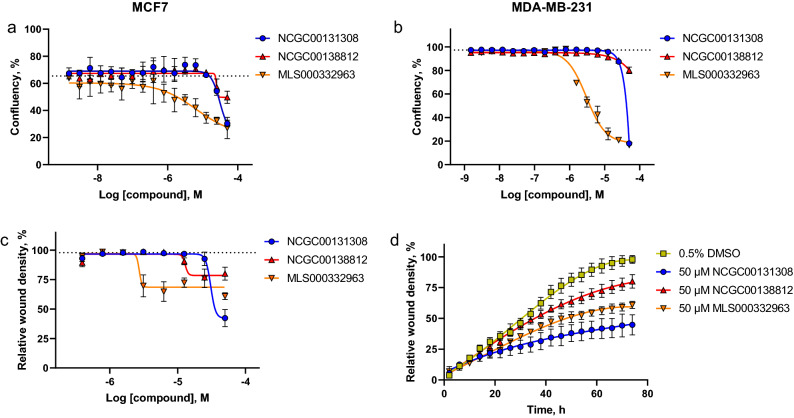
Dose-dependent effects of the selected compounds on cell proliferation and migration. MCF7 (**a**) and MDA-MB-231(**b**) cells were grown in the presence of compounds NCGC00131308, NCGC00138812 or MLS000332963 for 72 h and the confluency of the cell monolayer was measured. As control, cells were treated with 0.5% DMSO. The confluency of DMSO treated MCF7 cells was 65.47% ± 2.02 and MDA-MB-231 97.32% ± 0.84, respectively, and are depicted as dotted lines. (**c**,**d**) The migration of MDA-MB-231 cells was monitored in the presence of compounds NCGC00131308, NCGC00138812 or MLS000332963 at different concentrations using a scratch wound assay. (**c**) Concentration response analysis of the compounds at 72 h post treatment. The wound closure of DMSO treated control cells was 97.86% ± 3.17 and is depicted as a dotted line. (**d**) The effect of the compounds at 50 μM concentration on the migration of MDA-MB0231 cells over the course of treatment. Data are shown as means of triplicate measurements and standard deviations.

Because the interaction between IQGAP1 and Cdc42 enhances cell proliferation and migration, we examined if small molecules NCGC00131308, NCGC00138812 and MLS000332963 can modulate these functions in breast cancer cells. MCF7 and MDA-MB-231 cell growth was monitored in the presence of the compounds for 72 h using the IncuCyte live-cell analysis system. DMSO treated cells were used as the control. All three compounds had a dose-dependent inhibitory effect on proliferation of MCF7 and MDA-MB-231 cells (Fig. 5a,b).

MLS000332963 exhibited the highest potency among the tested compounds with EC_50_ values of 6.54 μM and 3.01 µM in MCF7 and MDA-MB-231 cells, respectively. The scratch wound assay was used to assess the effect of the compounds on cell migration of MDA-MB-231 cells. These cells were chosen for this assay because their migratory abilities are better than MCF7 cells. The cells were treated with NCGC00131308, NCGC00138812 and MLS000332963 molecules at different concentrations or with DMSO as control, and the migration of the cells was kinetically monitored for 72 h. All three compounds inhibited the cell mobility in a dose-dependent manner (Fig. 5c, Supplementary Fig. S2 online). The EC_50_ of MLS000332963 was 2.7 μM with maximum efficiency of 39%. NCGC00138812 was less active and had an EC_50_ of 12.9 µM and maximum efficiency of 20%. NCGC00131308 inhibited MDA-MB-231 cell migration by 57.7 ± 7.2% at 50 μM, the highest concentration used (Fig. 5d, Supplementary Fig. S2 online). Of note, MLS000332963 had an effect on the MDA-MB-231 cell shape as well. The cells became flatter and spread out more on the plastic (Supplementary Fig. S3 online).

### Microscale thermophoresis assay

**Figure 6:**
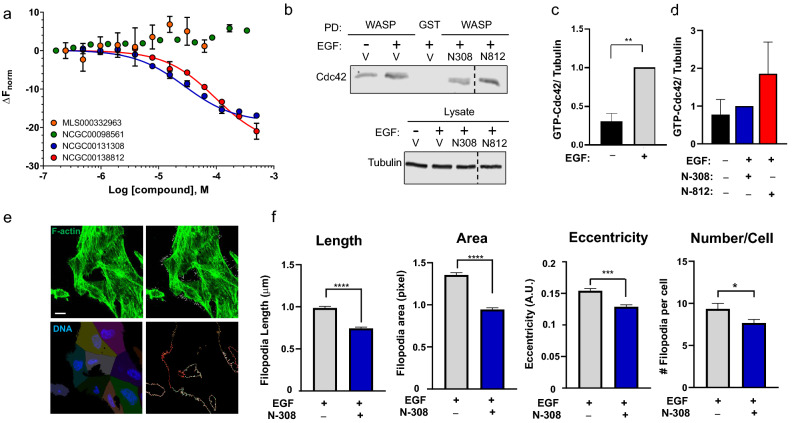
Interaction of selected compounds with Cdc42 and impact on Cdc42 activation. (**a**) Dose–response signals from microscale thermophoresis. Four compounds were titrated in 1:1 dilution series, starting at 500 µM (500 µM to 244 nM final concentration), and the normalized binding response (ΔFnorm) of each compound was calculated. MLS000332963 formed precipitates above 62.5 µM; these points were omitted from analysis. Data are means ± SD; n = 3. (**b**) MDA-MB-231 cells were serum starved for 16 h, then incubated with vehicle (V, DMSO), 50 µM NCGC00131308 (N308) or NCGC00138812 (N812). After 2 h, 100 ng/mL EGF ( +) or vehicle (-) was added for 10 min. After cell lysis, equal amounts of protein lysate were incubated with GST (control) or GST-WASP-GBD (WASP) for 2 h. Proteins were resolved by SDS-PAGE and probed with anti-Cdc42 antibody. Tubulin in lysates was the loading control. Dotted lines show where irrelevant lanes were removed from the blots. (**c**,**d**) The Cdc42 and tubulin bands were quantified and the amount of Cdc42 was corrected for tubulin in the same sample. Data are expressed as means ± SEM (n = 3). EGF-stimulated cells were set to 1 (**c**). NCGC00131308 (N308) was set to 1 (**d**). Vehicle treated samples are from the same experiments depicted in panel c (**e**) EGF-stimulated cells were stained with phalloidin (green) and Hoechst, then processed by confocal microscopy. White spikes depict individual filopodia (*upper right panel*). Cells were segmented by Voronoi tessellation using nuclei as seed points (*lower left panel*). Exposed cell membranes were segmented (*lower right panel*). Scale bar, 10 μm (**f**) Filopodia geometric parameters were quantified, including branch length (Length), cumulative area (Area), the ratio of minor and major axis length of the bounding box of each filopodial segment (Eccentricity), as well as number of filopodia per cell. At least fifty cells were analyzed. Data are expressed as means ± SEM (error bars) from three separate experiments, each conducted in triplicate. Unpaired *Student’s t-test*: * *p* < 0.05; ** *p* < 0.01 ***; *p* < 0.001; **** *p* < 0.001.

As mentioned above, the compounds NCGC00131308 and NCGC00138812 showed high activity in the HTRF counter-screen with the His and GST double-tagged Cdc42, but not in the counter-screen with His-tagged GST (Fig. 4c,d). These observations prompted us to investigate whether these compounds bind to Cdc42 rather than to IQGAP1 using the microscale thermophoresis assay. Additionally, we tested the compounds MLS000332963 and NCGC00098561, which were inactive in both counter-screens (Fig. 4a,b). Cdc42 exhibited negative thermophoresis in a dose-dependent manner in the presence of NCGC00131308 and NCGC00138812 (Fig. 6).

The dissociation constants (K_d_) for NCGC00131308 and NCGC00138812 were 28.12 µM ± 3.32 and 96.17 µM ± 14.41, respectively. In contrast, the small molecules MLS000332963 and NCGC00098561 did not affect the thermophoretic motion of Cdc42 (Fig. 6), suggesting that these compounds do not interact with Cdc42.

### Effects of the lead molecules on Cdc42 activation and filopodum formation

Based on the microscale thermophoresis assays, we selected compounds NCGC00131308 and NCGC00138812 for further cell-based studies. First, we evaluated the impact of the two compounds on the amount of active Cdc42. Serum starved MDA-MB-231 cells were incubated with DMSO (control), 50 μM NCGC00131308 or 50 μM NCGC00138812. After 2 h, cells were incubated with or without 100 ng/mL epidermal growth factor (EGF) for 10 min. The amounts of active Cdc42 in the cells were quantified using a GST-WASP-GBD pulldown assay. The WASP-GBD binds only GTP-bound (active) Cdc42; Therefore, the amount of bound Cdc42 indicates the amount of active Cdc42^19^. EGF significantly increases the amount of active Cdc42 in MDA-MB-231 cells incubated with DMSO (Fig. 6b,c). No Cdc42 bound to GST alone, documenting specificity (Fig. 6b). NCGC00131308 abrogated the increase in GTP-Cdc42 induced by EGF (Fig. 6b,d). By contrast, NCGC00138812 did not appreciably reduce activation of Cdc42 by EGF (Fig. 6b,d).

Changes in the levels of active Cdc42 directly impact actin cytoskeletal dynamics and the formation of filopodia^15,16^, which play an important role in adhesion and responses to external stimuli^17^. Therefore, we analyzed the effects of compound NCGC00131308 on the characteristics of filopodia in MDA-MB-231 cells. To visualize filopodia, we stained cells with phalloidin. The length, area, shape (eccentricity) and number of filopodia were quantified with FiloQuant FIJI (Fig. 6e,f). NCGC00131308 significantly decreased the length, area and the number of filopodia per cell (Fig. 6f), all of which directly results from defective actin polymerization at the site of filopodia formation^20^. In addition, NCGC00131308 altered the shape of the filopodia, inducing bending the filaments, which is evident by reduced eccentricity (Fig. 6f). Overall, our data reveal that compound NCGC00131308 reduces the amount of active Cdc42 induced by EGF in MDA-MB-231 cells, thereby reducing filopodia formation induced by EGF, a phenotype that affects migration of cancer cells^21^.

### Predicted binding model

To explore the binding interactions of NCGC00131308 and NCGC00138812 with Cdc42, we performed docking studies of these two molecules at the Cdc42-IQGAP1 binding interface and generated a possible binding model. Structural analysis of the PPI interface revealed a hydrophobic binding pocket adjacent to the GTP binding site of Cdc42, which is well-formed with potential for small molecule binding to disrupt the Cdc42-IQGAP1 interaction (Supplementary Fig. S4 online). The small molecules were therefore docked to the Cdc42 protein binding interface and the top-ranked docking poses were analyzed.

**Figure 7:**
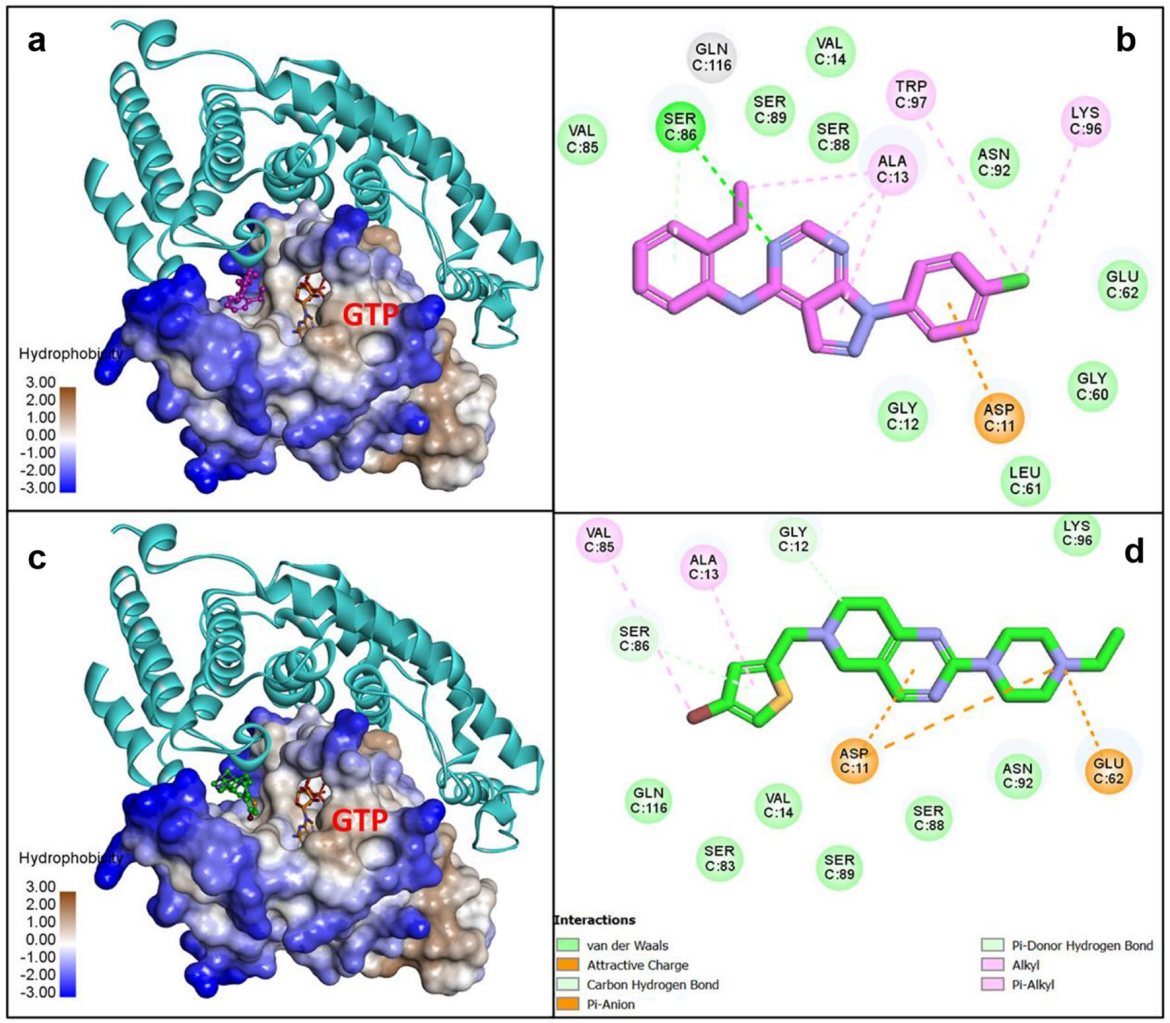
Predicted binding models of NCGC00138812 (**a**,**b**) and NCGC00131308 (**c** and **d**) bound to Cdc42 at the protein-protein interaction interface with IQGAP1. Cdc42 is shown in hydrophobic surface representation and IQGAP1 is shown as ribbons in cyan (**a**,**c**). GTP bound in Cdc42 is shown as sticks. 2D diagrams of binding interactions between Cdc42 and small molecule inhibitors are depicted in panels (**b**,**d**). The compounds are shown as sticks with carbon atoms in purple (NCGC00138812) and green (NCGC00131308). N and O atoms are colored blue and red, respectively. The figure was generated using the free open access software Discovery Studio Visualizer (https://discover.3ds.com/discovery-studio-visualizer-download).

The results showed that both inhibitors bind preferably to this pocket in a similar binding mode. As shown in the 2D diagram of inhibitor binding interaction analysis (Fig. 7), NCGC00131308 and NCGC00138812 fit in the pocket by forming extensive hydrophobic and aromatic interactions with surrounding residues A13, V85, S88, K96, and W97.

A hydrogen bonding interaction was formed with residue E62 and the piperazine ring of F2, while a hydrogen bonding interaction was formed between residue D11 and the pyrimidine ring of H3. In comparison, compounds MLS000332963 and NCGC00098561, did not make such key interactions in the pocket. These results may explain the MST assay results and provide structural insight as to how the small molecules are able to disrupt the interaction between Cdc42 and IQGAP1.

## Discussion

Interactions between proteins are central to both intracellular signaling and inter-cellular communications and regulate numerous fundamental biological processes. A single interface of a protein frequently interacts with several other proteins to mediate a wide array of interactions and associations in protein complexes^22^. The intricate protein–protein interactions are tightly regulated to drive specific signaling outcomes for the cells. In some pathological conditions, undesirable protein–protein interactions may occur, which may be important in the disease pathophysiology^23,24^. Small molecule inhibitors can effectively interrupt specific protein–protein interactions and can be used as therapeutic strategies^25^.

Cdc42 modulates numerous crucial physiological processes, such as cell cycle progression, cell migration, mitotic spindle orientation and establishment of polarity^26–28^. Additionally, Cdc42 is an important regulator of cytoskeletal dynamics during pathological conditions, such as invasion and metastasis of several types of cancer cells^29^, including breast carcinoma and metastatic melanoma^30,31^. Increased expression of Cdc42 correlates positively with cancer progression and poor clinical outcome in lung adenocarcinoma^32^, colorectal cancer^33^, breast carcinoma^34^ and melanoma^35^, which makes Cdc42 an appealing target for cancer therapy. Cdc42 functions are modulated by direct interactions with several effector and signaling proteins^36^. Therefore, efforts have been made to find small molecule inhibitors that can specifically disrupt the interactions of Cdc42 with selected signaling molecules to suppress selected cellular functions, such as motility and invasion^37^.

IQGAP1, a widely studied Cdc42 binding partner (for review, see Ref^4^), stabilizes Cdc42 in the active form^13^. Because overexpression of both IQGAP1 and Cdc42 is linked to carcinogenesis, small molecule inhibitors that efficiently disrupt the interaction between Cdc42 and IQGAP1 have potential for treating neoplastic progression and metastasis.

HTRF assays have been successfully used to screen and identify small molecules, which disrupt protein complexes and alter cell behavior^27^. This approach has been implemented to find new medications to prevent cancer cell invasion and metastasis^28^. Here we describe a novel high throughput HTRF assay to identify selective cell-active small molecule inhibitors that disrupt the interactions between Cdc42 and IQGAP1. We measured the molecular interactions between His-Cdc42 and GST-IQGAP1 using a pair of commercially available, fluorescent-tagged antibodies specific for His or GST. We thoroughly optimized the concentrations of recombinant proteins and antibodies, as well as binding buffer components, to obtain a high signal-to-background ratio (7.1-fold) and Z’-factor of 0.87, which render this assay suitable for HTS.

Eliminating false positive hits is a major challenge in HTRF assays^38^. To address this problem, we employed two different counter-screens. In the first, we directly linked the hexa-histidine and GST tags to each other. In the second, we developed a novel strategy to mimic the spatial architecture of the targeted protein complex and used dual-tagged His-Cdc42-GST. In both approaches, the fluorescent antibodies against the His and GST tags constantly stay in close proximity; only tag and/or antibody binders should show activity and would be considered as false positives.

We screened small molecule libraries of approximately 78,500 compounds and detected 198 compounds that significantly inhibited the interaction of Cdc42 with IQGAP1. These 198 compounds were tested in both counter-screens. The 44 most potent compounds (EC_50_ < 15 µM) from different chemotypes of structural clusters, which were inactive in the His-GST counter-screen, were selected for immunoprecipitation and cytotoxicity assays. Based on these assays, we selected four lead compounds, namely NCGC00131308, MLS000332963, NCGC00098561 and NCGC00138812, for further analysis. Importantly, all four compounds consistently impaired the interaction of Cdc42 with IQGAP1 in cell lysates.

From a medicinal chemistry perspective, these molecules have a reasonable structure for further structure–activity relationship and optimization studies. Especially appealing are the tetrahydropyrido pyrimidinamine NCGC00131308 and the pyrazolo pyrimidine NCGC00138812 compounds, which have desirable physicochemical characteristics, such as reasonable molecular weight, cLogP and tPSA, no chemically liable functionalities and an adequate number of hydrogen donors and acceptors. NCGC00098561, which showed good inhibitory activity in the biochemical HTRF and immunoprecipitation assays, had a high cytotoxicity. This cytotoxicity is probably independent of the targeted IQGAP1-Cdc42 interaction as the compound had very similar potency and efficacy in both control and IQGAP1-knockout MCF7 cells. Therefore, the compound was not tested further in our cell-based assays.

The intracellular interaction of Cdc42 with IQGAP1 promotes cell proliferation, motility and invasion^6,39^. Therefore, we evaluated the effect of NCGC00131308, MLS000332963, and NCGC00138812 compounds on the proliferation of MCF7 and MDA-MB-231 human breast carcinoma cells as well as on migration of MDA-MB-231 cells. All three compounds effectively inhibited cell growth and migration, suggesting that they modulate cellular processes by interfering with IQGAP1-Cdc42 interactions.

Interestingly, NCGC00131308, MLS000332963 and NCGC00138812 behaved similarly in all assays, except the counter-screen with the dual-tagged Cdc42. NCGC00131308 and NCGC00138812 inhibited the HTRF signal in a dose-dependent manner, while MLS000332963 was inactive. We hypothesized that NCGC00131308 and NCGC00138812 might bind to Cdc42 and disrupt the homodimerization of Cdc42 induced by the dimerization of GST^40^. The second counter-screen with His-tagged GST indicates that none of the selected compounds interacts with antibodies to hexa-histidine, GST and/or HTRF because they were inactive in this assay. Also, we used the same concentrations of His-GST and His-Cdc42-GST (100 nM) in both counter-screens, so, if existent, the monomer–dimer equilibrium in both assays is comparable.

Analysis by microscale thermophoresis revealed that compounds NCGC00131308 and NCGC00138812 interact directly with Cdc42. By contrast, MLS000332963 and NCGC00098561 do not bind. NCGC00131308 prevented the increase in active Cdc42 induced by EGF in breast carcinoma cells. Active Cdc42 drives the formation of straight and stable filopodia, which confer adequate force to push the membrane edge during directional migration^41^. To ascertain whether the inhibitors impair Cdc42-dependent cellular effects, we analyzed filopodia formation by confocal microscopy. Consistent with the results of active Cdc42 assays, NCGC00131308 significantly decreased the length and number of filopodia generated by EGF. Moreover, NCGC00131308 causes bent filopodia with less eccentricity, which indicates reduced filopodia stiffness and likely contributes to decreased directional cell migration in our wound assay.

To gain additional insight into how the four lead compounds suppress the interaction between Cdc42 and IQGAP1, we performed computational modeling of the Cdc42 interface interacting with these compounds. Docking analysis provided plausible models of compounds NCGC00131308 and NCGC00138812 binding to the hydrophobic pocket adjacent to the GTP binding site of Cdc42 at the IQGAP1 interface, which could disrupt the Cdc42-IQGAP1 interaction. Computational modeling showed that compounds MLS000332963 and NCGC00098561 do not fit well in the hydrophobic pocket, consistent with our experimental observations by thermophoresis.

Accumulating evidence suggest that small GTPases, such as Rab and Ras, dimerize to function as signaling units with the capacity to activate cellular cascades, including the mitogen-activated protein kinase and PI3K/Akt pathways^42,43^. Understanding the biology of small GTPase dimerization has profound implications for pharmacological targeting of signaling pathways in cancer. We previously solved the crystal structure of Cdc42 bound to the GRD of IQGAP2, which revealed that four molecules of Cdc42 bind two IQGAP2 molecules^12^. Importantly, two Cdc42 molecules form a dimer in the complex^12^. Elucidating the mechanism of action of the four compounds that we identified requires additional investigation. However, it is tempting to speculate that the small molecule inhibitors interfere with dimerization of Cdc42 molecules at the IQGAP1 binding interface^44^.

The interaction of IQGAP1 with Cdc42 regulates the invasion of *Salmonella typhimurium*^45^ and other bacteria^46^ into host cells. IQGAP1 also participates in the pathogenesis of viral infections^47^. Thus, small molecule inhibitors of the Cdc42/IQGAP1 interaction could potentially be used against invading microorganisms.

In conclusion, we developed a novel HTRF-based strategy and identified four small molecule inhibitors that disrupt the interaction of Cdc42 with IQGAP1. The approach we developed could also be used to identify inhibitors that interfere with the association of Cdc42 with other binding partners to develop new therapies against neoplastic transformation and other pathological conditions, such as microbial infections.

## Methods

### Materials

Anti-6HIS-XL66 (#61HISXLB) and anti-GST-cryptate (#61GSTKLB) were purchased from Cisbio Bioassays, recombinant His-tagged GST (#12-523) from Millipore. Monolith NT.115 Instrument, Monolith His-Tag Labeling Kit RED-Tris-NTA & Monolith NT.115 standard capillaries were from Nanotemper Technologies, Munich, Germany. Anti-IQGAP1 polyclonal antibodies have been characterized previously^48^. Mouse monoclonal anti-Cdc42 antibodies were purchased from Santa Cruz (#sc-8401). IRDye® -800CW and -680RD (Life Technologies TM, USA) were used as secondary antibodies for Western blotting at 1:10,000 dilutions.

### Constructs and recombinant proteins

The generation of both the C-terminal half of IQGAP1 (comprising amino acids 864–1657) containing a GST-tag^48^ and His-Cdc42-Q61L^44^ have been described previously. The His-Cdc42-Q61L construct was generated from pGEX-Cdc42-Q61L (residues 1–184 of Cdc42; generously donated by Darerca Owen, University of Cambridge, Cambridge, UK) by excising the plasmid and inserting it into pRSET. To make dual-tagged GST-Cdc42-Q61L-His, a pGEX4T-His vector was generated. For this purpose, the phosphorylated oligo pair with nine His tags were annealed and inserted into pGEX4T at XmaI and XhoI sites (5′-CCGGGCTGGAAGTTCTGTTCCAGGGGCCCCATCATCATCATCATCATCATCATCATCAT-3′;5′-TCGAGATGATGATGATGATGATGATGATGATGATGGGGCCCCTGGAACAGAACTTCCAG-3′). To make pGEX4T-Cdc42-Q61L-His, Cdc42-Q61L was synthesized by PCR using pGEX2T-Cdc42-Q61L as template with the forward primer 5-CGGGATCCCAGACAATTAAGTGTGTTGTTGTGGG-3′; and reverse primer 5'-CCGGAATTCTTAGAATATACAGCACTTCC-3’. Synthesized Cdc42 was cut with BamH I and EcoR I and inserted into pGEX4T-His at BamH I and EcoR I sites. DNA sequencing confirmed the sequences of all constructs.

GST-IQGAP1-C and GST-Cdc42-Q61L-His were expressed in *Escherichia coli* BL-21 and isolated using glutathione-Sepharose chromatography, essentially as previously described^48^. His-Cdc42-Q61L was expressed in *E. coli* and purified by nickel-immobilized metal affinity chromatography as previously described^44^.

### Primary HTRF Assay for High Throughput Screening (HTS)

Recombinant His-Cdc42-Q61L, GST-IQGAP1-C in combination with acceptor XL665 conjugated anti-His antibody (Anti-His-XL665) and donor Eu-cryptate conjugated anti-GST antibody (anti-GST-cryptate) were used for the HTRF assay to perform a high throughput screening of compound libraries in 1536-well white solid bottom medium-binding plates (Greiner Bio-One).

For the assay, His-Cdc42-Q61L and GST-IQGAP1-C proteins were diluted and pre-mixed in the binding buffer (50 mM Tris, pH 7.5, 150 mM NaCl and 0.01% Triton-X100) to final assay concentrations of 50 nM His-Cdc42-Q61L and 1.5 nM GST-IQGAP1-C. As control, 1.5 nM GST-IQGAP1-C in the absence of His-Cdc42-Q61L was used to gauge the maximal potential inhibition (IC_100_). Anti-His-XL665 and anti-GST-cryptate antibodies were prepared as threefold concentrated working mixture in binding buffer just prior to dispensing, with final assay concentrations of 5 ng/μL and 0.7 ng/µL, respectively. Pre-mixed proteins (4 µL/well) were dispensed to assay plates with a BioRAPTR FRD™ dispenser. The compounds, dissolved in DMSO, were pin-transferred to the wells (23 nL/well) with a Kalypsus pintool instrument (Wako Automation), followed by addition of 2 µL/well pre-mixed antibodies. The plates were incubated for 3 h at ambient temperature. Then, 1 μL/well of 2.8 M potassium fluoride was dispensed to prevent possible quenching, i.e., to stabilize the signal. The signal was detected immediately with an Envision plate reader (Perkin Elmer) using the HTRF settings (excitation at 340 nm, dual emission at 665 nm and 620 nm). DMSO alone was used as the vehicle control (IC_0_). The data were calculated as the ratio 665/620 × 10^4^ to normalize for any effects in the donor channel. The assay protocol is outlined in Supplementary Table S1 online.

A 1536-well plate with the established assay conditions and chosen controls of DMSO as IC_0_ and 1.5 nM IQGAP1 protein as IC_100_ was used to establish the signal-to-background (S/B) ratio and Z’ factor characteristics of the assay. Then, the Library of Pharmacologically Active compounds (LOPAC^®1280^, Sigma Aldrich) collection was screened in dose response for the purpose of establishing the robustness of the assay in a screening format and in the presence of the compounds. Compounds titrated 1:5 at seven concentrations in an interpolation manner were tested. The final concentrations of the compounds in the assay ranged from 38 µM to 3.6 nM. Following assay validation, the primary high-throughput screening HTS was performed with multiple NCATS in-house screening collections, at four final concentrations (38 µM to 105 nM).

Inhibitors of the IQGAP1/Cdc42 HTRF binding assay from the primary HTS were selected using compound dose response curve algorithms developed at NCATS, which assigns a curve response classification (CRC) number to each tested compound^18^. This method classifies primary hits into different categories according to their potency (IC_50_), magnitude of response (efficacy), quality of curve fitting (r2), and number of asymptotes. Selected compounds were re-tested using the HTRF assay to confirm initial activity, following the same format described above in a dose-dependent manner at seven concentrations in 1:3 serial dilutions.

### HTRF counter-screens

Two different HTRF counter-screens were used to identify false positive hits. The two interacting proteins GST-IQGAP1-C and His-Cdc42-Q61L were replaced with His-tagged GST or Cdc42-Q61L containing GST at the N-terminus and His at the C-terminus (GST-Cdc42-Q61L-His).

First, GST-Cdc42-Q61L-His was titrated in parallel with the pre-mixed His-Cdc42-Q61L and GST-IQGAP1-C to comparatively match the signal level to the signal of the primary HTRF assay. We chose 100 nM GST-Cdc42-Q61L-His protein as the final concentration for the counter-screen. The assay protocol is outlined in Supplementary Table S2 online. Briefly, 4 μL/well of GST-Cdc42-Q61L-GST diluted in binding buffer was dispensed to the plates, followed by pinning 23 nL/well of selected hit compounds from the primary screening and addition of 2 μL/well premixed HTRF antibodies. After 3 h, 1 μL/well of 2.8 M potassium fluoride was added to the assay and the fluorescence signal was read on the Envision instrument. For controls, we used pre-mixed proteins GST-IQGAP1-C and His-Cdc42-Q61L (primary assay’s regular conditions) as IC_100_, and His-Cdc42-Q61L only at 1.5 nM final concentration as IC_0_.

The second counter-screen was performed with 100 nM His-tagged GST following the protocol described above.

### Cell culture

Cells were grown in Dulbecco's modified Eagle's medium (DMEM, ATCC#30-2002) supplemented with 10% (v/v) fetal bovine serum (FBS) (ATCC# 30-2020). MCF7 cells with stable knockdown of IQGAP1 (termed MCF-siIQ8) and control MCF7 cells stably expressing Renilla have been described previously^39^. Briefly, a stable IQGAP1-deficient MCF7 cell line was generated by integrating a specific siRNA targeted against IQGAP1 into the genome of MCF7 malignant breast epithelial cells^39^. The protein expression of IQGAP1 in knockdown cells is 80% lower than in parent MCF-7 cells^39^. HEK293, MCF7 and MDA-MB-231 cells were purchased from ATCC.

### CellTiter-Glo® luminescent cell viability assay

Selected hit compounds were tested in CellTiterGlo cell viability assays to determine their cytotoxic effect on IQGAP1 knockout and control MCF7 cells. Prior to the assay, we assessed cell proliferation to keep both cell lines at similar exponential growth rates within 80 h after plating. For this purpose, the cells were plated onto 384-well clear bottom black plates (Perkin Elmer, Ultra) at various cell densities (between 625 and 10,000 cells/50µL/well), spun down for 15 s, and placed into Incucyte SX5 (Essen BioScience) for automated bright-field channel reading every 4 h. Based on the growth curve, the densities for the two cell lines that displayed comparable exponential growth within 75 h were selected and the seeding number adjusted to be suitable for the 1536-well plate. For the cytotoxicity assays, cells were harvested with 0.05% trypsin, spun down at 125x*g* for 5 min, re-suspended in fresh DMEM supplemented with 5% (v/v) FBS, and seeded into 1536-well white solid bottom tissue culture-treated plates (Greiner One, #789173-F) at 600 cells/5µL/well for knockout cells and 300 cells/5µL/well for control cells using a Multidrop Combi dispenser (Thermo Fisher Scientific Inc.). One column on each plate was dispensed with growth medium only (no cells) to gauge the maximal potential cytotoxic effect (IC_100_ control). The plates were kept in a tissue culture incubator at 37 °C with 5% CO_2_ for ~ 20 h for attachment and recovery of the cells. The next day, selected compounds were pin-transferred into the wells in columns 5–48, 23nL/well at 7-point 1:3 serial dilution, in the range of 46 µM to 78 nM final assay concentration. DMSO was used as the vehicle control (IC_0_). The plates were covered with stainless steel cell culture Kalypsys lids and incubated at 37 °C with 5% CO_2_ under 95% humidity for 72 h.

Plates and reagents were equilibrated to room temperature and then 5 µL/well of CellTiter Glo (Promega, #G7570) reagent was dispensed with a Multidrop Combi dispenser to the assay plates. After incubation for 10 min at ambient temperature, the luminescence signal was read with a ViewLux instrument (Perkin Elmer) with one second exposure. Relative luminescent units (RLU) for each well were normalized to the median RLUs from DMSO control wells as 0% and “no cells” control wells as 100% viability.

### Proliferation assay

MDA-MB-231 and MCF7 cells were grown to confluence, detached using trypsin–EDTA cell dissociation buffer (Gibco 12604013) and counted. Cells were plated into 384-well, clear-bottom, black, tissue culture treated plates (Corning 3683) using an automatic dispenser (Thermo Multidrop Combi SMART) at densities of 2000 cells/10 µL/well for MDA-MB-231 cells and 4000 cells/10 µL/well for MCF7 cells in DMEM supplemented with 5% FBS. After 4 h incubation at 37 °C with 5% CO_2_, the growth medium was replaced with 10 µL medium containing compounds at 16-point 1:2 serial dilutions with the final highest concentration of 50 µM for each compound. All samples containing compounds were plated in triplicate. Cells in the first column of the plate were treated with 0.5% DMSO as control. The plates were placed into an Incucyte SX5 Live-cell imaging system (Sartorius) and phase images were taken at 6-h intervals for 72 h with 10X objective lens. The growth medium containing compounds or DMSO was replenished after 24 h. The images were analyzed, and the growth curves generated by the Incucyte software 2020A using the basic analyzer mode.

### Scratch wound migration assay

MDA-MB-231 cells were grown to confluence and plated into a 96-well clear plate (Sartorius 4379) at 30,000 cells/100 µL/well in DMEM supplemented with 5% FBS. Cells were incubated at 37 °C with 5% CO_2_ overnight. Even wounds were created using a 96-pin wound device (Sartorius 4493) following the manufacturer’s protocol. Medium was then aspirated, and the cells were washed once with PBS pH 7.4 before adding 100 µL/well of fresh growth medium. Then 100 µL/well of growth medium containing compounds at an 8-point 1:2 titration dispensed into the cell plate resulting a final highest concentration of 50 µM for each compound. 0.5% DMSO was used as control. All samples were plated in triplicate. The plate was placed into an Incucyte SX5 Live-cell imaging system and phase images were taken in 2-h intervals for 72 h using the scratch wound setting. Medium was aspirated and replaced with a fresh medium containing compounds or DMSO after 24 h. The images were analyzed, and the wound density curves generated by the Incucyte software 2020A using the scratch wound analysis option.

### Microscopy and image analysis

MDA-MB231 cells were cultured in 24-well plates containing glass coverslips. When 60% confluent, cells were serum starved for 16 h, preincubated with 50 μM compound NCGC00131308 for 2 h, then incubated with 100 ng/mL epidermal growth factor (EGF) or vehicle for 10 min. Cells were fixed with 4% PFA and stained with Alexa Fluor 488 Phalloidin (Invitrogen™#A12379) and Hoechst 33342 (BD Biosciences #561908) to visualize F-actin and DNA, respectively. Coverslips were mounted with ProLong glass antifade mounting medium (Invitrogen), then imaged with a Zeiss LSM880 confocal microscope using a 63× objective lens. Cells and filopodia were segmented using FIJI/ImageJ^49^. Individual filopodia were segmented using the FiloQuant FIJI plugin^50^. Cells were segmented by Voronoi tessellation using Hoechst 33342 stained nuclei as seed points. The segmented images were quantified using the *scikit-image* Python package^51^. The following geometric and spatial parameters of the filopodia were quantified: (1) the branch length of each filopodial segment, (2) cumulative area of each segment, (3) eccentricity, defined as the ratio of the minor axis length to the major axis length, and (4) the number of filopodial segments per unit exposed cell membrane. Further data handling and plotting were done using the tidyverse R package. At least 200 cells were analyzed for each condition.

### Immunoprecipitation and western blotting

HEK293 cells were plated in 10-cm dishes to reach 80% confluence. The following day, the cells were washed with ice-cold phosphate-buffered saline (PBS) and lysed with 500 µl of Buffer A (50 mM Tris–HCl, pH 7.4, 150 mM NaCl, and 1% Triton X-100) supplemented with complete protease and phosphatase inhibitors (Roche, Mannheim, Germany). Lysates were subjected to two rounds of sonication for 10 s each, and insoluble material was precipitated by centrifugation at 13,000 xg for 10 min at 4 °C. Supernatants were precleared with protein A-Sepharose beads for 1 h. DMSO (vehicle) or different concentrations of the compounds (0.2, 0.6, 1, 5, 16 or 50 µM) were added to equal amounts of protein lysates for 30 min. Protein A-Sepharose beads and anti-IQGAP1 polyclonal antibodies were then added for 2 h at 4 °C. Anti-rabbit IgG antibodies were used as the immunoprecipitation control. Samples were washed five times with Buffer A, resolved by SDS-PAGE and transferred to polyvinylidene fluoride (PVDF) membranes (Millipore Corp). Membranes were probed with anti-IQGAP1 and anti-Cdc42 antibodies. After secondary antibody detection, Western blots were imaged using a LI-COR Odyssey infrared imaging system (LI-COR, USA). Western blot quantification and analysis was conducted using Image Studio™ Lite (LI-COR Biosciences).

### Assay of active Cdc42

The GST-WASP-GBD (GTPase-binding-domain) has been described previously^19^. Briefly, GST-WASP-GBD was generated by polymerase chain reaction, digested, and ligated into a pGEX-KG vector^52^. Following expression of the GST fusion protein in *E. coli*, purification was performed by glutathione-Sepharose affinity chromatography. MDA-MB-231 cells were grown in DMEM with 10% FBS to 60% confluence. Cells were starved of serum for 16 h, then incubated with either DMSO (vehicle) or 50 μM of the compounds indicated in the figure legend. After 2 h, 100 ng/mL EGF or vehicle was added for 10 min. After washing with 1xPBS, cells were lysed in lysis buffer (20 mM Hepes, pH 7.4, 150 mM NaCl, 1% NP-40, 20 mM NaF, and 20uM GTP, containing halt protease & phosphatase inhibitor cocktail (Thermo Scientific #1,861,281). Lysates were precleared with glutathione-Sepharose for 1 h at 4 °C and equal amounts of protein was incubated with either GST (control) or GST-WASP-GBD for 2 h at 4 °C. Complexes were collected with glutathione-Sepharose and washed 5× with lysis buffer. Bound proteins were resolved by SDS-PAGE and transferred to a polyvinylidene difluoride membrane. The membrane was cut immediately below the 37 kDa molecular weight marker. The top portion of the membrane was discarded and the bottom portion was probed with anti-Cdc42 antibody (BD Transduction Laboratories #610929). Aliquots of whole cell lysate were processed in parallel. The membrane was cut immediately below the 37 kDa molecular weight marker. After transfer, the bottom portion of the membrane was discarded and the top portion was probed with anti-tubulin (Sigma–Aldrich #T5201) antibody (loading control). The intensities of the Cdc42 and tubulin bands were quantified using Image Studio (LI-COR Biosciences).

### Microscale thermophoresis (MST)

The interactions between Cdc42 and selected compounds were evaluated by MST using His-Cdc42 fluorescently labeled with Monolith His-tag labeling RED-Tris-NTA 2nd Generation kit (Nanotemper Technologies) following the manufacturer’s protocol. Two-fold serial dilutions of compounds were first prepared in DMSO, then further diluted in PBS containing 0.05% Tween-20. Compounds were incubated with an equal volume of 100 nM labeled Cdc42 for 5 min at room temperature. Samples were loaded into Monolith™ standard capillaries and measured using Monolith NT.115 (Nanotemper Technologies) with 50% excitation power and 50% MST power, with laser on and off times of 30 s and 5 s, respectively. The K_d_ values were calculated by evaluating the thermophoresis and T-Jump signals using MO.Affinity Analysis software (Nanotemper Technologies). All experiments were conducted in three biological replicates.

### Molecule docking

The crystal structures of IQGAP1 in apo form (PDB code 3FAY)^53^ and IQGAP2 bound to Cdc42 (PDB code 5CJP)^12^ have been solved. Therefore, we generated the binding complex of IQGAP1/Cdc42 by superimposing IQGAP1 to IQGAP2 in the crystal structural complex with Cdc42. The binding model of IQGAP1/Cdc42 was used to predict inhibitor binding interaction. Docking studies were performed using the MOE program^54^.The ligand induced fit protocol was used and the binding affinity was evaluated using the GBVI/WSA score.

### Statistical analyses

All data are representative of at least three independent experiments conducted with three technical replicates unless indicated otherwise in the figure legends. Statistical analyses were performed using GraphPad Prism software (version 8.0; San Diego, CA). Bars indicate mean ± standard errors.

## Supplementary Information

Supplementary Information.

## Data Availability

All data generated or analysed during this study are included in this published article [and its supplementary information files]. The study includes no data deposited in external repositories.

## References

[1] Briggs MW, Sacks DB (2003). IQGAP proteins are integral components of cytoskeletal regulation. EMBO Rep..

[2] Hedman AC, Smith JM, Sacks DB (2015). The biology of IQGAP proteins: Beyond the cytoskeleton. EMBO Rep..

[3] Choi S, Hedman AC, Sayedyahossein S, Thapa N, Sacks DB, Anderson RA (2016). Agonist-stimulated phosphatidylinositol-3,4,5-trisphosphate generation by scaffolded phosphoinositide kinases. Nat. Cell Biol..

[4] Smith JM, Hedman AC, Sacks DB (2015). IQGAPs choreograph cellular signaling from the membrane to the nucleus. Trends Cell Biol..

[5] White CD, Brown MD, Sacks DB (2009). IQGAPs in cancer: A family of scaffold proteins underlying tumorigenesis. FEBS Lett..

[6] Jadeski L, Mataraza JM, Jeong HW, Li ZG, Sacks DB (2008). IQGAP1 stimulates proliferation and enhances tumorigenesis of human breast epithelial cells. J. Biol. Chem..

[7] Li SH, Wang QJ, Chakladar A, Bronson RT, Bernards A (2000). Gastric hyperplasia in mice lacking the putative Cdc42 effector IQGAP1. Mol. Cell Biol..

[8] Jameson KL (2013). IQGAP1 scaffold-kinase interaction blockade selectively targets RAS-MAP kinase-driven tumors. Nat. Med..

[9] Svensmark JH, Brakebusch C (2019). Rho GTPases in cancer: Friend or foe?. Oncogene.

[10] Bourne HR, Sanders DA, Mccormick F (1990). The Gtpase superfamily—A conserved switch for diverse cell functions. Nature.

[11] Hart MJ, Callow MG, Souza B, Polakis P (1996). IQGAP1, a calmodulin-binding protein with a rasGAP-related domain, is a potential effector for cdc42Hs. EMBO J..

[12] LeCour L, Boyapati VK, Liu J, Li Z, Sacks DB, Worthylake DK (2016). The structural basis for Cdc42-induced dimerization of IQGAPs. Structure.

[13] Swart-Mataraza JM, Li ZG, Sacks DB (2002). IQGAP1 is a component of Cdc42 signaling to the cytoskeleton. J. Biol. Chem..

[14] Owen D (2008). The IQGAP1-Rac1 and IQGAP1-Cdc42 interactions -Interfaces differ between the complexes. J. Biol. Chem..

[15] Nobes CD, Hall A (1995). Rho, rac, and cdc42 GTPases regulate the assembly of multimolecular focal complexes associated with actin stress fibers, lamellipodia, and filopodia. Cell.

[16] Kozma R, Ahmed S, Best A, Lim L (1995). The ras-related protein Cdc42Hs and bradykinin promote formation of peripheral actin microspikes and filopodia in swiss 3T3 fibroblasts. Mol. Cell Biol..

[17] Machesky LM, Hall A (1997). Role of actin polymerization and adhesion to extracellular matrix in rac- and rho-induced cytoskeletal reorganization. J. Cell Biol..

[18] Inglese J (2006). Quantitative high-throughput screening: A titration-based approach that efficiently identifies biological activities in large chemical libraries. Proc. Natl. Acad. Sci. USA.

[19] Kim SH, Li Z, Sacks DB (2000). E-cadherin-mediated cell-cell attachment activates Cdc42. J. Biol. Chem..

[20] Schafer DA (2004). Cell biology: Barbed ends rule. Nature.

[21] Arjonen A, Kaukonen R, Ivaska J (2011). Filopodia and adhesion in cancer cell motility. Cell Adhes. Migr..

[22] Tonddast-Navaei S, Skolnick J (2015). Are protein-protein interfaces special regions on a protein's surface?. J. Chem. Phys..

[23] Kann MG (2007). Protein interactions and disease: Computational approaches to uncover the etiology of diseases. Brief. Bioinform..

[24] Ideker T, Sharan R (2008). Protein networks in disease. Genome Res..

[25] Garner AL, Janda KD (2011). Protein-protein interactions and cancer: Targeting the central dogma. Curr. Top. Med. Chem..

[26] Schachterle C, Christian F, Fernandes JM, Klussmann E (2015). Screening for small molecule disruptors of AKAP-PKA interactions. Methods Mol. Biol..

[27] Basu S (2019). Design, synthesis, evaluation, and structural studies of C2-symmetric small molecule inhibitors of programmed cell death-1/Programmed death-ligand 1 protein–protein interaction. J. Med. Chem..

[28] Dai X (2017). Aspirin inhibits cancer metastasis and angiogenesis via targeting heparanase. Clin. Cancer Res..

[29] Stengel K, Zheng Y (2011). Cdc42 in oncogenic transformation, invasion, and tumorigenesis. Cell. Signal..

[30] Philip R (2000). Dendritic cells loaded with MART-1 peptide or infected with adenoviral construct are functionally equivalent in the induction of tumor-specific cytotoxic T lymphocyte responses in patients with melanoma. J. Immunother..

[31] Zhang Y, Li J, Lai XN, Jiao XQ, Xiong JP, Xiong LX (2019). Focus on Cdc42 in breast cancer: new insights, target therapy development and non-coding RNAs. Cells.

[32] Gomez Del Pulgar T (2008). Cdc42 is highly expressed in colorectal adenocarcinoma and downregulates ID4 through an epigenetic mechanism. Int. J. Oncol..

[33] Liu Y (2013). Abnormal expression of Pygopus 2 correlates with a malignant phenotype in human lung cancer. BMC Cancer.

[34] Fritz G, Brachetti C, Bahlmann F, Schmidt M, Kaina B (2002). Rho GTPases in human breast tumours: Expression and mutation analyses and correlation with clinical parameters. Br. J. Cancer.

[35] Tucci MG (2007). Involvement of E-cadherin, beta-catenin, Cdc42 and CXCR4 in the progression and prognosis of cutaneous melanoma. Br. J. Dermatol..

[36] Melendez J, Grogg M, Zheng Y (2011). Signaling role of Cdc42 in regulating mammalian physiology. J. Biol. Chem..

[37] Friesland A, Zhao Y, Chen YH, Wang L, Zhou H, Lu Q (2013). Small molecule targeting Cdc42-intersectin interaction disrupts golgi organization and suppresses cell motility. Proc. Natl. Acad. Sci. USA.

[38] Zhang JH, Wu X, Sills MA (2005). Probing the primary screening efficiency by multiple replicate testing: A quantitative analysis of hit confirmation and false screening results of a biochemical assay. J. Biomol. Screen..

[39] Mataraza JM, Briggs MW, Li ZG, Entwistle A, Ridley AJ, Sacks DB (2003). IQGAP1 promotes cell motility and invasion. J. Biol. Chem..

[40] Maru Y, Afar DE, Witte ON, Shibuya M (1996). The dimerization property of glutathione S-transferase partially reactivates Bcr-Abl lacking the oligomerization domain. J. Biol. Chem..

[41] Clayton NS, Ridley AJ (2020). Targeting Rho GTPase signaling networks in cancer. Front. Cell Dev. Biol..

[42] Daitoku H, Isida J, Fujiwara K, Nakajima T, Fukamizu A (2001). Dimerization of small GTPase Rab5. Int. J. Mol. Med..

[43] Nan X (2015). Ras-GTP dimers activate the mitogen-activated protein kinase (MAPK) pathway. Proc. Natl. Acad. Sci. USA.

[44] Ozdemir ES (2018). Unraveling the molecular mechanism of interactions of the Rho GTPases Cdc42 and Rac1 with the scaffolding protein IQGAP2. J. Biol. Chem..

[45] Brown MD, Bry L, Li Z, Sacks DB (2007). IQGAP1 regulates salmonella invasion through interactions with actin, Rac1, and Cdc42. J. Biol. Chem..

[46] Kim H, White CD, Sacks DB (2011). IQGAP1 in microbial pathogenesis: Targeting the actin cytoskeleton. FEBS Lett..

[47] Leung J, Yueh A, Appah FS, Yuan B, de los Santos K, Goff SP (2006). Interaction of Moloney murine leukemia virus matrix protein with IQGAP. EMBO J..

[48] Ho YD, Joyal JL, Li ZG, Sacks DB (1999). IQGAP1 integrates Ca2+/calmodulin and Cdc42 signaling. J. Biol. Chem..

[49] Schindelin J (2012). Fiji: An open-source platform for biological-image analysis. Nat. Methods.

[50] Jacquemet G (2017). FiloQuant reveals increased filopodia density during breast cancer progression. J. Cell Biol..

[51] van der Walt S (2014). scikit-image: Image processing in Python. PeerJ.

[52] Zhang B, Wang ZX, Zheng Y (1997). Characterization of the interactions between the small GTPase Cdc42 and its GTPase-activating proteins and putative effectors. Comparison of kinetic properties of Cdc42 binding to the Cdc42-interactive domains. J. Biol. Chem..

[53] Kurella VB, Richard JM, Parke CL, Lecour LF, Bellamy HD, Worthylake DK (2009). Crystal structure of the GTPase-activating protein-related domain from IQGAP1. J. Biol. Chem..

[54] Molecular Operating Environment (MOE). Chemical Computing Group ULC (2018).

