# Supplementary material for: Discovery of small molecule inhibitors that effectively disrupt IQGAP1-Cdc42 interaction in breast cancer cells
Source: Sci Rep. 2022 Oct 17;12:17372. doi: 10.1038/s41598-022-21342-w (PMC9576799; doi:10.1038/s41598-022-21342-w)
Supplement: Supplementary file 1 — Supplementary Information. [file 41598_2022_21342_MOESM1_ESM.pdf]

Supplementary information

Fig. S1: The effect of DMSO on the assay signal of the Cdc42/IQGAP1 HTRF assay. HTRF assay depicting signal intensity in samples containing either no His-Cdc42, no DMSO or 4% DMSO in the reaction mixture. The Y-axis represents the time resolved fluorescence detected at 615 and 665 nm. The ratio of 665/615x10^4 was plotted. Data were obtained from n=32 replicates of no His-Cdc42, n=128 of no DMSO and n=26 of 4% DMSO.

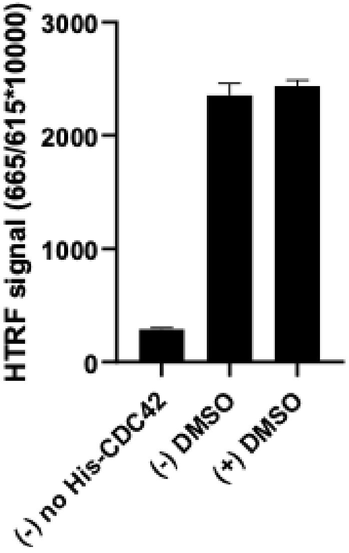

Fig. S2: Effect of the compounds on the migration of breast carcinoma cells. Representative images of scratch wound assay conducted with MDA-MB-231 cells at specified time points. Scale, 500 µm.

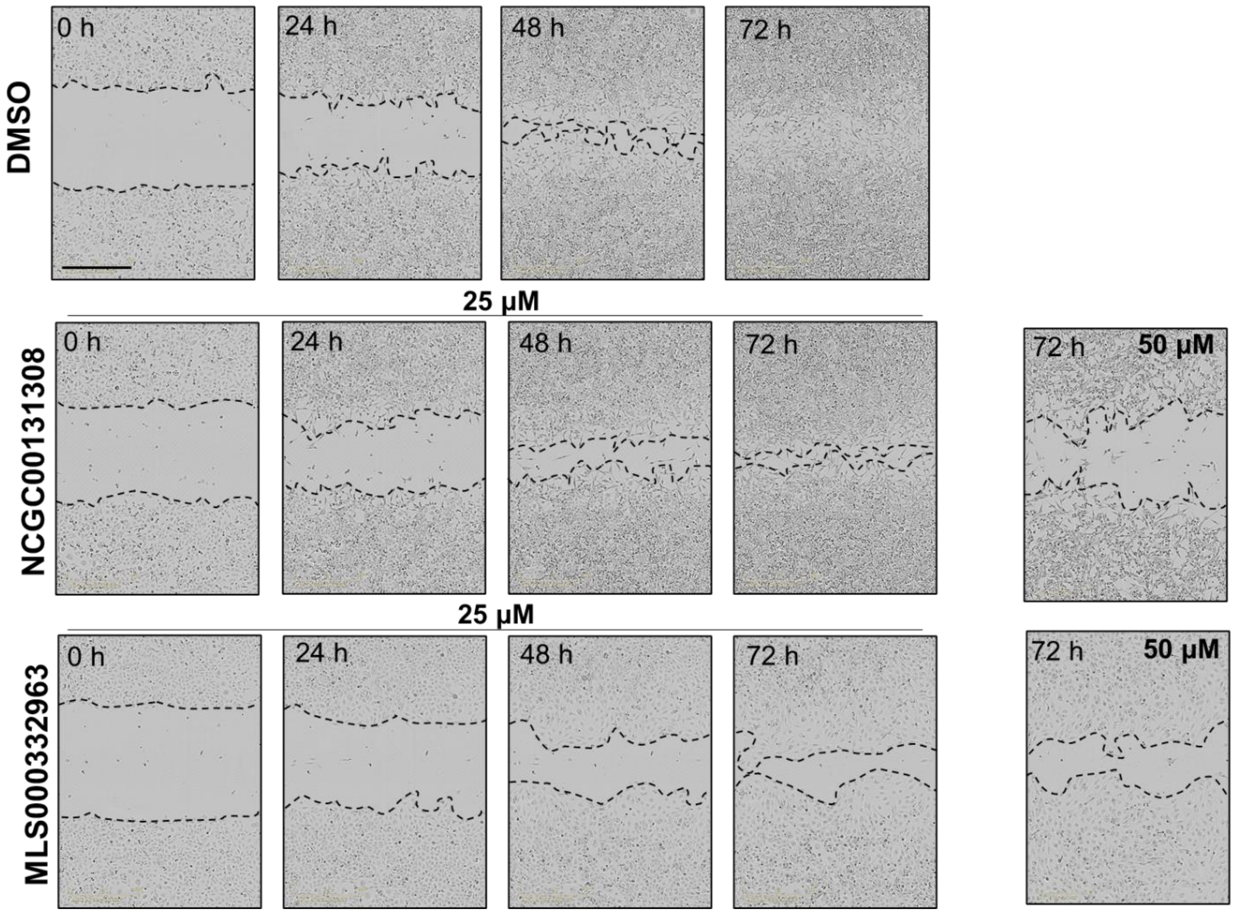

Fig. S3: Effect of the MLS000332963 compound on the morphology of MDA-MB-231 cells.

Representative images of DMSO (vehicle control) and compound (50 µM) treated cells. Scale, 100 µm.

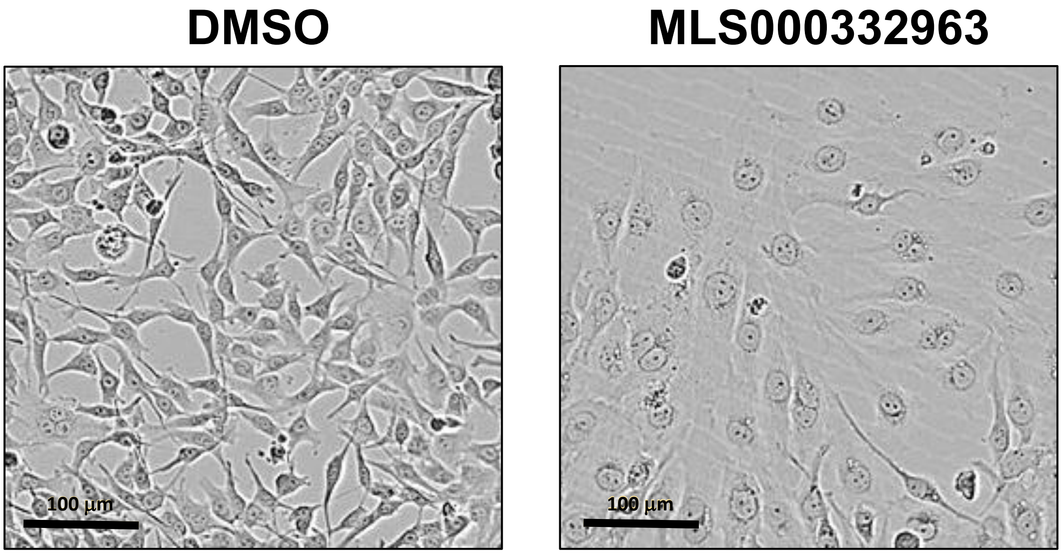

Fig. S4: Structural analysis of the Cdc42-IQGAP1 binding interface. MOE program was used to generate the docking model. Cdc42 is shown in hydrophobic surface representation. The dashed yellow line indicates the position of small molecule (PPI inhibitor) adjacent to the GTP binding pocket. The figure was generated using open access Discovery Studio Visualizer (https://discover.3ds.com/discovery-studio-visualizer-download).

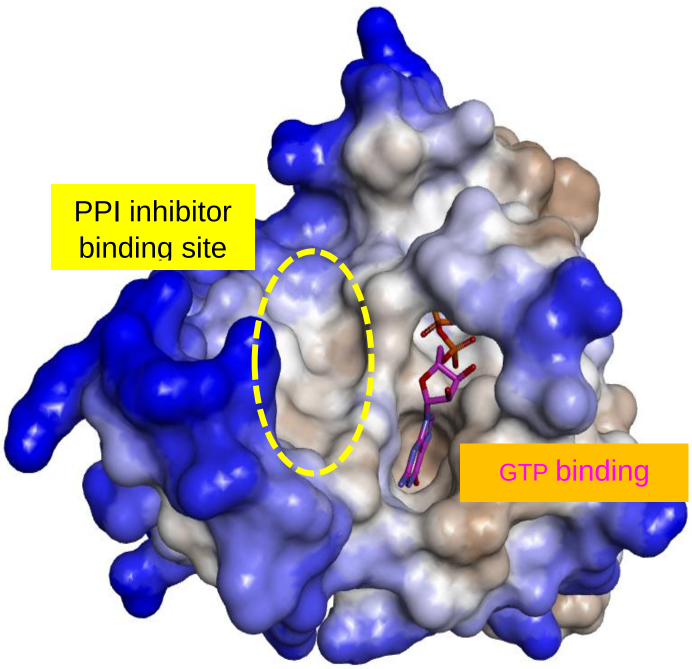

Fig S5: Raw images of Fig. 1b

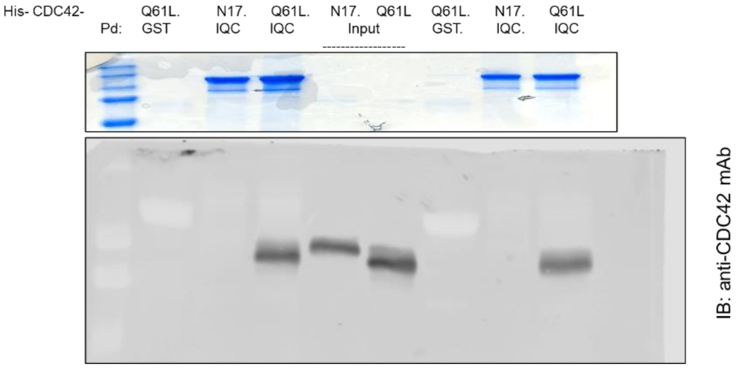

Different exposures of the blot on the left

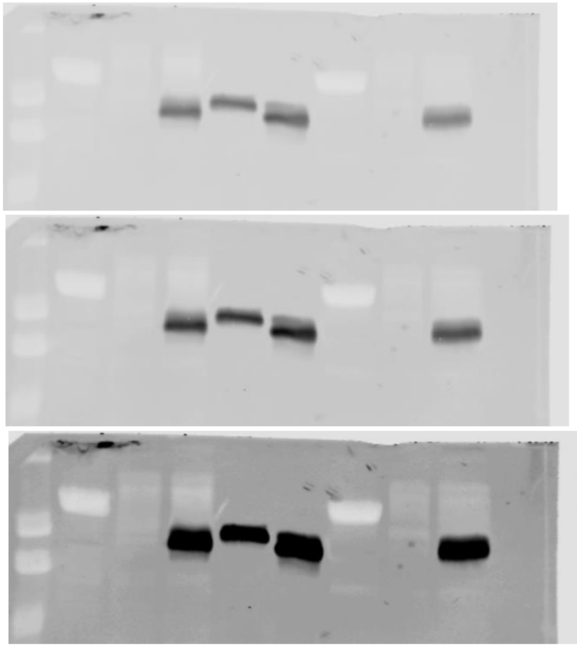

Fig. S6: Raw images of Fig. 3a

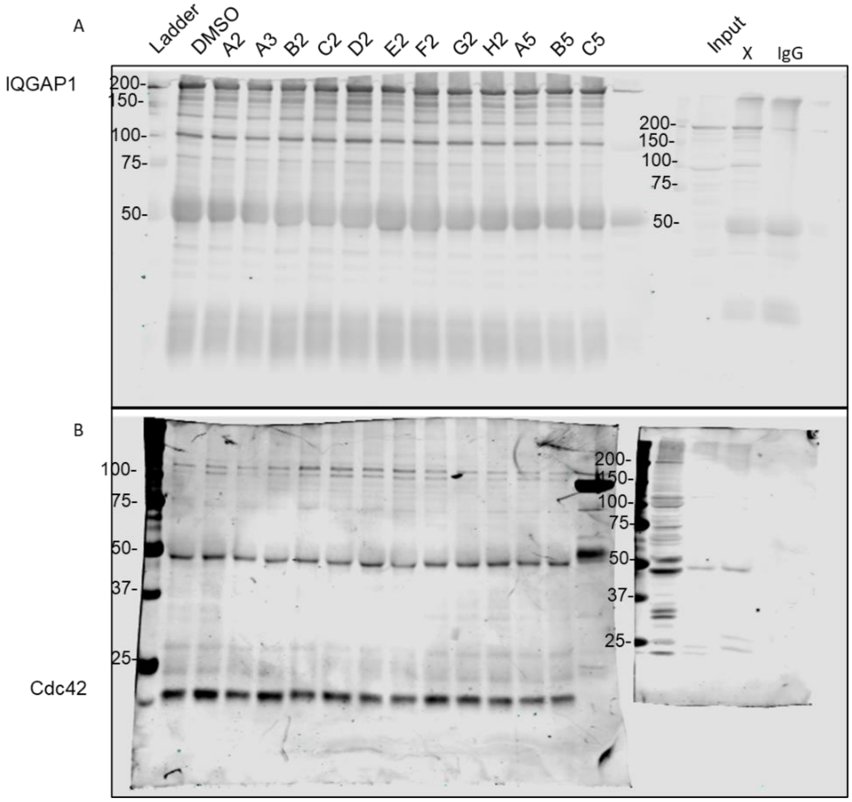

Different exposures of the blot on the left

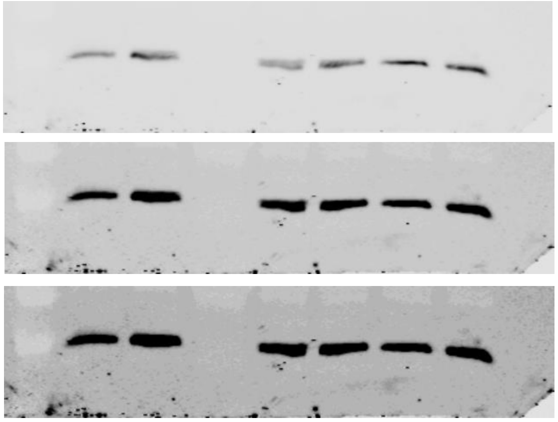

Fig S7: Raw images of Fig. 6b

Repeat#1

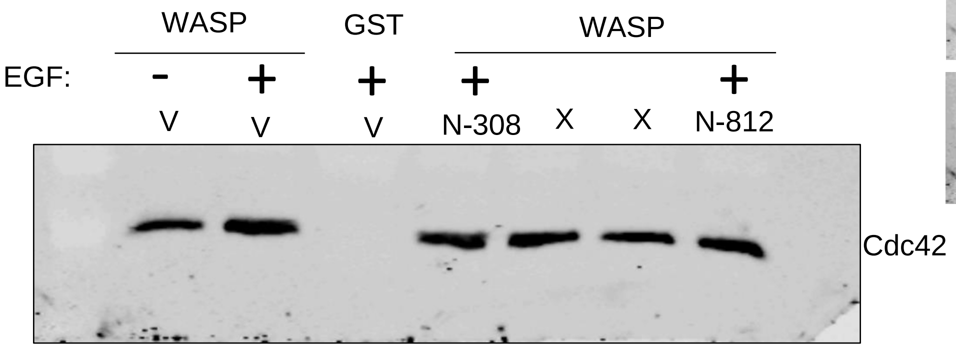

Different exposures of the blot on the left

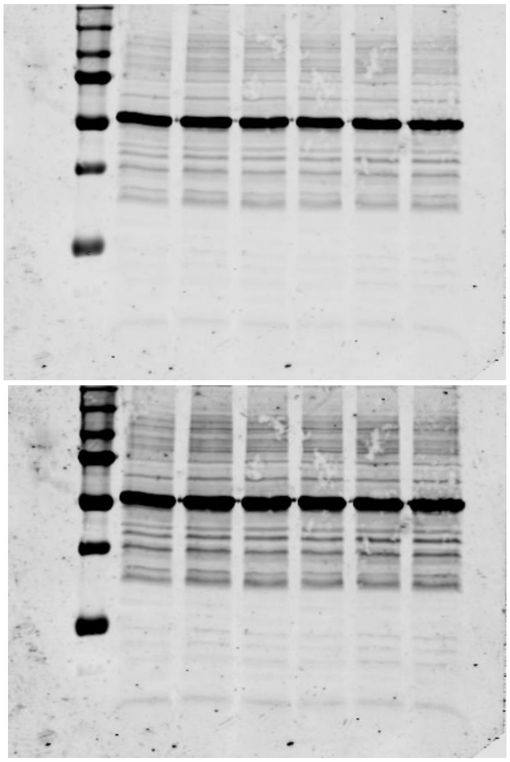

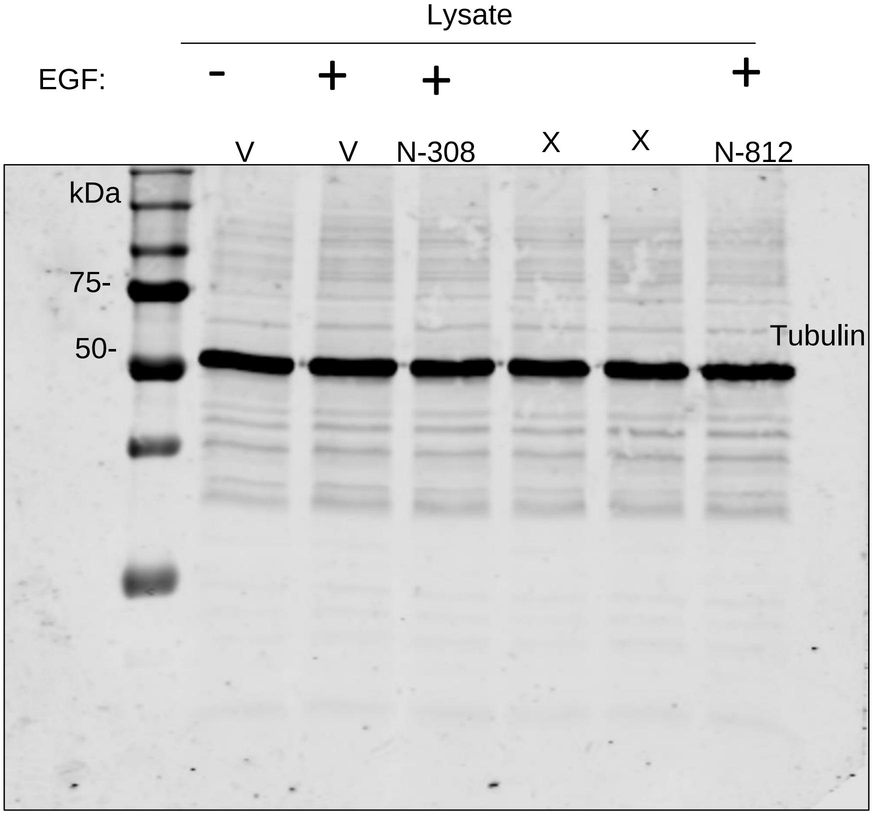

Repeat#2

Different exposures of the blot on the left

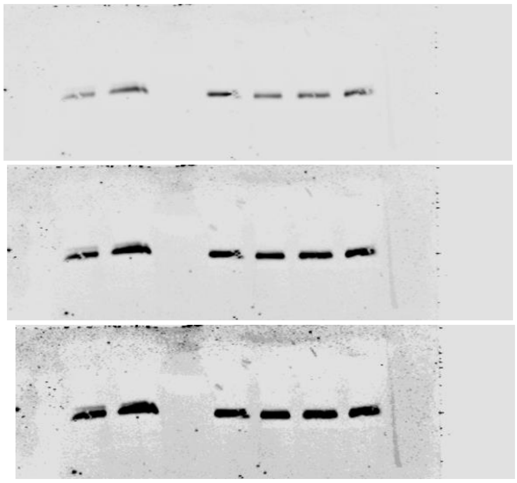

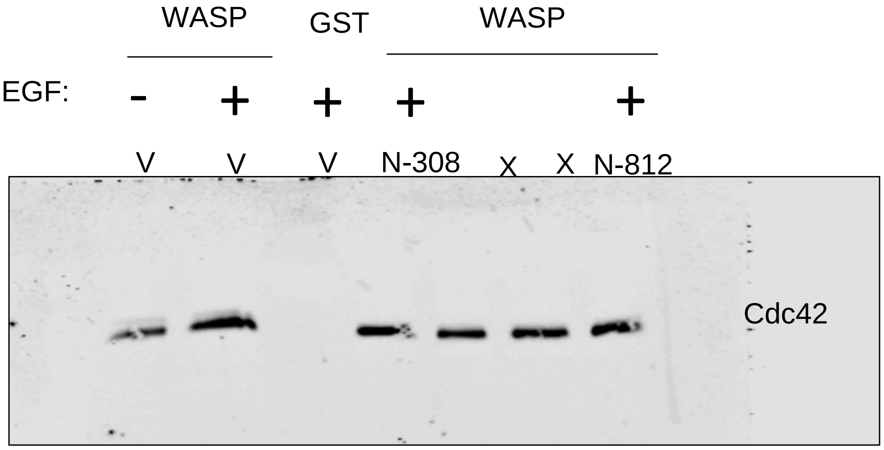

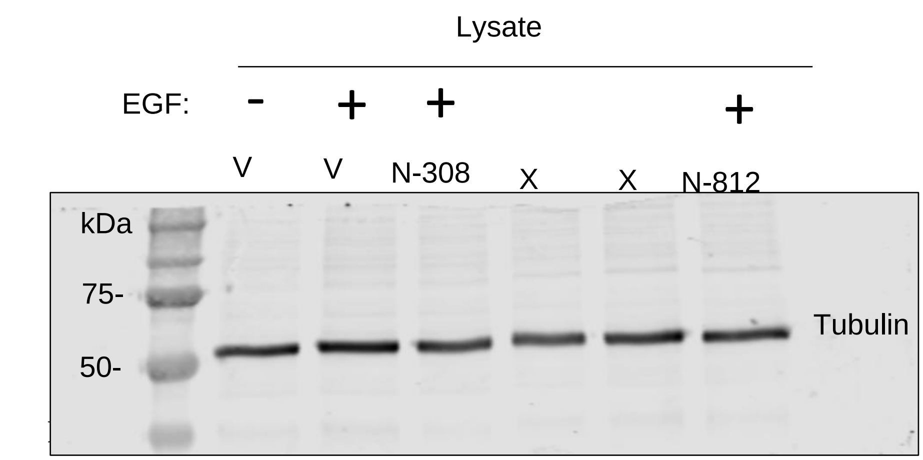

Different exposures of the blot on the left

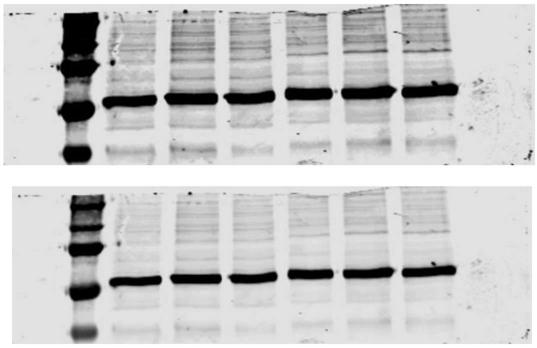

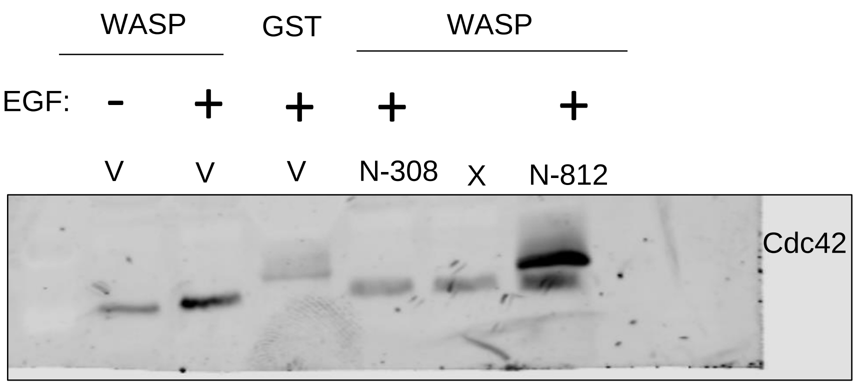

Different exposures of the blot on the left

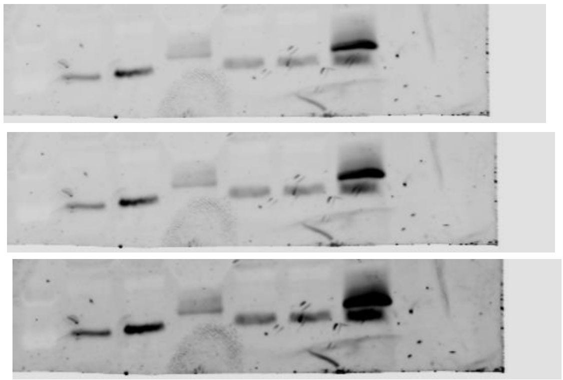

Different exposures of the blot on the left

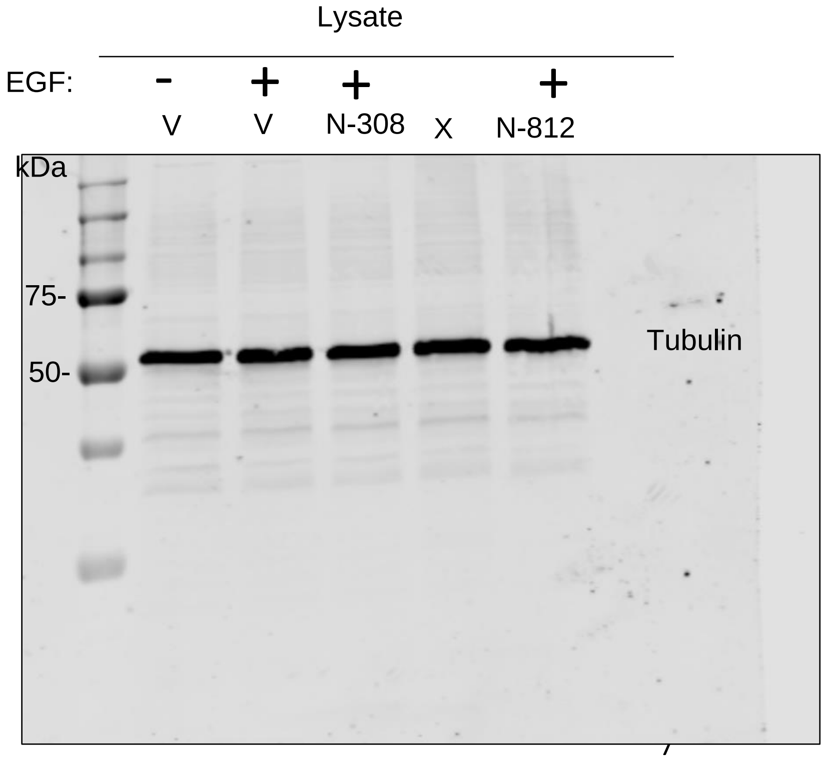

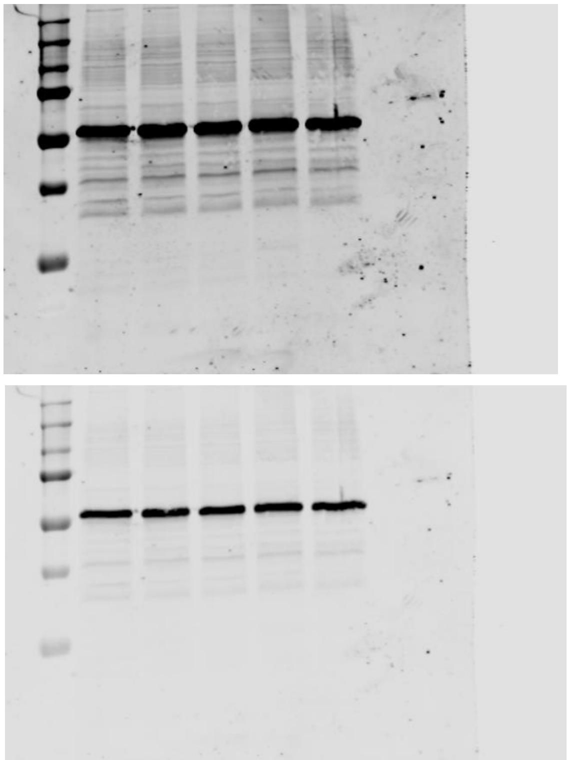

Supplementary Table S1. IQGAP1-Cdc42 binding HTRF Assay Protocol.

Step	Parameter	Value	Description
1a	GST-IQGAP1-C and His-Cdc42-Q61L premix	4 µL	**Columns 1-2, 4-48**<br>50nM His-Cdc42-Q61L +<br>1.5nM GST-IQGAP1-C (final concentrations), pre-mixed in binding buffer
1b	GST-IQGAP1-C protein only	4 µL	**Column 3:** IC_{100} control, 1.5nM final concentration
2a<br>2b	Compounds Library or Control	23 nL	Column 1 – DMSO as IC_0 control
3	HTRF antibodies pre-mix	2 µL	α-His-XL (5 ng/µL, 3X working concentration) +<br>α-GST-K (0.7 ng/µL 3X working concentration), in binding buffer
4	Time	3 hours	Incubation (ambient temperature)
5	Reagent	1 µL	2.8 M KF (7x)
6	Detector	HTRF	Envision plate reader (excitation: 340 nm, emission: 620 and 665 nm)

Supplementary Table S2: Counter-screening HTRF protocol.

Step	Parameter	Value	Description
1a	Protein	4 µL	Columns 4-48: 100 nM His-Cdc42-GST or<br>100 nM His-GST (final concentration), diluted in binding buffer
1b	Controls	4 µL	Column 1: buffer only<br>Column 2: GST-IQGAP1-C protein only [1.5nM] final<br>Column 3: pre-mixed proteins (regular assay conditions)
2	Compounds	23 nL	Selected compounds were pin-transferred from DMSO stock
3	HTRF antibodies pre-mix	2 µL	α-His-XL (5 ng/µL, 3X working concentration) +<br>α-GST-K (0.7 ng/µL 3X working concentration), mixed in binding buffer
4	Time	3 hours	Incubation (ambient temperature)
5	Reagent	1 µL	2.8 M KF (7x)
6	Detector	HTRF	Envision plate reader (excitation: 340 nm, emission: 620 and 665 nm)
